# Navigating through the maze of pricing and affordability of branded pharmaceuticals in the midst of the financial crisis: a comparative study among five European recession countries, from a Cyprus perspective

**DOI:** 10.1186/s40545-016-0057-7

**Published:** 2016-03-15

**Authors:** Panagiotis Petrou, Michael A. Talias

**Affiliations:** Open University of Cyprus, Nicosia, Cyprus

**Keywords:** Affordability, Financial recession, Branded pharmaceuticals, Cyprus, Italy, Spain, Portugal, Greece

## Abstract

**Background:**

Financial recession mandated the introduction of harsh austerity measures. Health, and particularly pharmaceuticals, constitute a significant part of public expenditure and as such they have been subject to significant budget reduction and stringent policies. As a consequence of these measures, an increasing percentage of patients resort to private sector for acquisition of their prescribed pharmaceuticals, due to exclusion of public health care beneficiary status, reduction of breadth of national formularies, delays in reimbursement and excessive waiting times. Affordability for pharmaceuticals in the private sector is of paramount importance since household disposable income plummets and more people are prone to impoverishment. This is critical for branded products, whose active substance and trademark are under patent protection, since no alternative options exist while their monopoly status imply that their prices are high. The impact on affordability regarding access of patient to necessary pharmaceutical care has not been documented in developed countries.

**Methods:**

A laspeyer index was constructed to compare prices of branded pharmaceuticals and assess affordability, by adjusting price index with Gross Domestic Product Purchase Power Parity per capita. Laspeyer index compares prices based on weights, which in our study are the corresponding sales of products in Cyprus. Moreover, we define the percentage of population that will face catastrophic pharmaceutical expenditure after acquisition of one product from eight major and common therapeutic categories. We used data from five European recession countries: Italy, Portugal, Spain, Greece and Cyprus, for 48 products which were selected based on sales.

**Results:**

Cyprus displays the highest prices for pharmaceuticals. By adjusting for Gross Domestic Product Purchase Power Parity per capita, affordability is worst for Cyprus and Portugal.

**Conclusions:**

As more patients have to resort to private sector for provision of adequate and timely healthcare, health agencies must reassess affordability of medicines and minimise catastrophic expenditure impact. Health agencies should primarily try to enhance efficiency of the system and reduce waste, instead of resorting to blunt budget reduction, which can demonstrate unpredictable consequences in public health.

## Key points

Financial recession exerts significant pressure on public expenditure and disposable household income.

Crisis and austerity measures compromised the functional capacity of public health sector and more people have to resort to private health care sector for timely care.

Affordability of branded pharmaceuticals in the private sector surfaces as a significant element, since it may impede access of patients to necessary medicines.

Among 5 Mediterranean EU countries in recession, the affordability for Cypriot patients is in the lowest position.

Governments should closely monitor affordability issue of medicines and distribute financial burden equally among all involved parties.

## Background

In 2007 the worst financial crisis, after the Great Depression in the 30s, emerged and quickly escalated into a global concern. In 2009, European Union’s Gross Domestic Product (GDP) subsided by 4.9 % indicating the impact across world’s second economy [1]. Reduced GDP ensued to reduction of tax revenues, while unemployment and fall of household disposable income increased demand for counterpoising public resources in health and social care.

Impaired funding capacity had to satisfy increased needs. Health agencies tried to increase their efficiency and adjust breadth, scope and depth of health care coverage [2]. A general perception is that latter policies are, by default, deprived of long-term vision and focus on short-term savings [3]. Fiscal balance and adherence to public expenditure target is acknowledged as the primary goal during fiscal crisis since these are the decisive factors for disbursement of financial instalments by international lenders. This unilateral focus on public expenditure [4] explains why the majorities of measures dealt with public sector, while private sector was largely overlooked since expenditure is out-of-pocket and therefore not directly interrelated with fiscal deficit.

Private health expenditure during crisis is also massively impaired. Primarily, crisis reduced household disposable income, compromising ability to pay and signalling a shift of patients to public health care sector. At the same time, public health expenditure resources are reduced as unemployment and salary reductions take their toll on tax income. We must underline that crisis, both at macro (unemployment) and micro level (job loss), is an independent risk factor for several health conditions which increases health care demand and Health Agencies end up balancing between the conflicting tasks of financing increased needs with reduced funds. If more funds are allocated to health sector, this will inflict losses on public expenditure and consequently it will deteriorate fiscal deficit ultimately, this will spare-out resources from other publicly funded programs, such as social welfare and education, while fiscal deficit was acknowledged as an independent health risk factor.

In the pharmaceutical sector, changes have occurred with a dramatically fast pace. Although the exact pattern varies across countries, there are more similarities than disparities and the broad framework resembles among them (Table 1). This is expressed in increase of personal contribution and copayment, exclusion of products from formulary, monitoring of prescribing, introduction of Health Technology Assessment (HTA), and reduction of refill duration to avoid waste. Elasticity of income can classify medicines as a necessity [5] and evidence from previous financial crisis showed that, in general, consumption of medicines was not compromised during economic crisis.

**Table 1:** Importance of private sector during financial crisis

Reasons	Countries affected
Exclusion of many patients from public health coverage	Spain, Greece, Cyprus, Portugal
Extended freeze of inclusion of new products in the formulary	Spain, Greece, Cyprus, Italy, Portugal
Exclusion of several products from formulary	Spain, Greece, Cyprus, Italy, Portugal
Prolonged shortages of reimbursed medicines	Spain, Greece, Italy, Portugal
Long waiting lists in the public sector due to austerity measures (recruitment freeze of health professionals)	Spain, Greece, Cyprus, Italy, Portugal

During the 90’s crisis in Asia, the decline in consumption of medicines lagged GDP decline [6]. The consumption of medicines for chronic diseases recovered quicker than overall consumption, while the consumption for acute health conditions recovered slower than GDP. These findings refer to low income countries, which demonstrate inordinate pharmaceutical prices compared to average income, pharmaceutical expenditure represents a significant percentage of total health expenditure and, most importantly, health insurance coverage is not universal. In the Baltics, crisis was associated with a significant reduction in the sales of over-the-counter pharmaceutical products (OTC-pharmaceutical products which can be dispensed without a prescription), while among prescription products only the consumption of pscyhoanaleptic products demonstrated reduction [6]. European Health systems offer a universal health coverage, which renders a safety net for patient through its prepayment mechanism for risk pooling. A recent study among EU countries reported an increase in pharmaceutical sales volumes almost in all of them regardless fiscal position, while value of sales declined, with a more pronounced effect in recession counties, which is attributed to more frequent and steeper price reduction [7].

Despite universal access pattern of EU healthcare systems, it is anticipated that a steadily increasing number of patients rely on out-of-pocket payment (private sector) for their necessary health care [8] (Table 2). To begin with, exclusion of many former beneficiaries from public health coverage occurred, both direct and indirect. Direct exclusion of beneficiaries was observed due to the detrimental outcome of policies, which were primarily applied to target some of the causes of recession, such as tax evasion. Specifically this led to exclusion of many unemployed from public coverage as following:

**Table 2:** Cost-containment measures implemented by selected countries

	Portugal	Greece	Spain	Cyprus	Italy
Savings for the payer (Health System for Portugal, Greece, Spain, Italy- Private Sector’s patients in Cyprus )	343 million euros (−11.7 %) IN 2012	1 billion euro in 2012. (from €5.4 billion in 2010 to an estimated 3.5 in 2012)	8.8 % reduction in 2011 pharmaceutical expenditure for 2011	8.5 % Reduction for 2015	25.1 billion in 2012 to $23.5 billion in 2020 – a decrease of $1.6 billion in eight years. [http://www.pharmaphorum.com/articles/the-current-healthcare-regulatory-and-reimbursement-landscape-in-italy]
Pharmacists	Mark-up profit reduction	Mark-up profit reduction Introduction of Rebates/clawback	Mark-up profit reduction	Introduction of regressive mark up profit plus fee for service	Mark –up profit reduction
Pricing	7 % average price cut on drugs	25 % temporary price cuts reduction, Regular Price interventions	Lowest Price among EU	External reference pricing through one expensive, one cheap and two medium priced EU countries	Renegotiation of the prices of less effective medicines
			Up to 30 % price reduction for medicines in 2010		
	6 % mandatory discount in retail price for all reimbursed medicines				
	20–35 % price cut for some generics products				
	7.5 % price cut for biologics				
Generics	Incentives for generic prescribing. Priced 50 % below the RRP of the reference product, or 25 % per cent if the wholesale price is less than €10)	Obligatory generic penetration at least 40 % of medicines used in public hospitals	INN prescribing	N/A	12.5 % reduction in the prices of generic. Generic penetration is 20 % (volume).
			(Royal Decree 16/2012) Obligatory dispensing of the cheapest generic version of a drug.		
		Pricing cannot exceed 40 % of the equivalent branded product.			
		60 % of value of prescribed products must be generics			
Prescribing	INN prescribing	INN prescribing	INN prescribing	INN prescribing only in public sector	INN prescribing
		Doctors have a personal budget for pharmaceuticals equals to 80 % of corresponding last year’s period.			
User Charges	Increase of contribution	1 euro medical prescription fee 0 %	1€ rate per prescription. Annual cap independent on income Pensioners: co-payment rate 10 % of Price with monthly cap depending on income		Co-payment Increase (varying regional levels)
	Tier A, 90 % of the public price of the drug is reimbursed. This tier is for essential drugs to treat severe diseases;				
		10–25 % personal contribution. Only cheapest generic product is reimbursed			
			Changes in co-payment rates for non-pensioners: 40, 50 or 60 % depending on income. No cap		
	Tier B: 69 % of the public price is reimbursed (essential drugs for chronic diseases)				
	Tier C: 37 % of the public price is reimbursed. (drugs which have a confirmed therapeutic interest)				
	Tier D: 15 % of the public price is reimbursed. (new medicines)				
Reimbursement	Delay of medicine’s reimbursement	Delay of medicine’s reimbursement	Dispensing of medicines for a maximum period of 30 days. (chronic conditions are exempted) 417 medicines indicated for minor symptoms are excluded		Reimbursements prices for generic drugs to the average European level.
					Regular Revisions the list of reimbursable pharmaceuticals
Efficiency gains	Centralised hospital drug purchasing system	Tendering	Tendering	HTA (Public sector only)	Regulation of Length of stay Reduction of hospital beds
		HTA	Economic evaluation	Clinical Pathways (Public sector only)	
		DRG			
		Electronic Prescribing			
		Charge for hospital admissions [37].			
					Definition of admission criteria
					Monitor of pharmaceuticals consumption across regions
					Strick controls over hospital budgets for pharmaceuticals
					Centralisation of procurement procedures HTA electronic medical records
					Pay-for-performance schemes
			HTA		
	Electronic prescribing				
	Guidelines Increase of patients assigned to GP				
Industry	Clawback	Clawback	Clawback	N/A	Pharmaceutical expenditure ceiling to13 percent of total health expenditure. Overall pharmaceutical spending cannot exceed 16 percent of health expenditure [12].
			Additionally 15 % rebate on products marketed for more than 10 years but with no equivalent generic or biosimilar in the market		
Cost Reduction	Breadth	Breadth	Breadth	Breadth	Breadth
	Scope	Scope	Scope	Scope	Scope
	Depth of health coverage	Depth of health coverage	Depth of health coverage	Depth of health coverage	Depth of health coverage

- Unemployed Cypriots must have contributed to social insurance for three years before they are entitled to free medical care. Therefore, newly recruited employees are not covered.
- Unemployed Spanish citizens over 26 are entitled only to emergency care.
- Unemployed people in Greece are also excluded.

Moreover, sudden impoverishment and reduction of household’s disposable income forced a significant percentage of patients in Greece to forfeit their health insurance fees, in order to satisfy more urgent needs, such as housing. In addition to the above, other traits of the post-recession healthcare market, as formed by recession and austerity measures, shift indirectly patients to private sector. This is induced by:

- Prolonged freeze of inclusion of new products in the formulary therefore patients can procure the product only through private sector.
- Exclusion of several former reimbursed products from formularies due to reduction of their breath, which leads to distortion of continuity of care and forces patients to continue buying the product in the private sector, and
- Prolonged shortages of reimbursed medicines.

Other cost-containment approaches include the reimbursement of only a single product from a broader therapeutic category, which is procured through tendering, an aggressive pricing and reimbursement scheme. This leads to significant savings for the buyer, since high and guaranteed estimated sales volume facilitates greater price reduction through enhanced competition. Nevertheless, this may backfire in case patients do not respond or tolerate the specific product. Under these circumstances, people resort to the private sector and all costs incur patient. Furthermore, in the broader health sector, long waiting lists, ascribed to austerity measures, such as recruitment freeze of health professionals further aggravate public health, forcing people to private sector, especially for acute conditions and time sensitive health conditions.

Current literature lacks affordability studies on behalf of private sectors patients, which is attributed to the theoretical universal coverage that EU health system provide to all EU citizens. Affordability is, to a certain extent, an ambiguous term [9, 10]. It usually describes establishing and maintaining an optimum (as perceived by the individual taking into consideration contextual particularities of each country and socioeconomic environment) living standard, which is compatible to an individual's income. Therefore, affordability is intertwined with the income of each household, price of relevant product and exact description of the “unreasonable financial burden”. Foremost, it is massively impaired when health expenditure exceeds 40 % of the total income of the individual, and any health expenditure higher than this, are considered to be catastrophic. For medicines alone, this percentage is 5 % [11].

The scope of this paper is to assess the impact of financial recession on prices and affordability of branded products among 5 EU countries that are experiencing financial recession.

## Methods

### Data

This study was performed from a Cyprus perspective, using sales volume from Cyprus pharmaceutical market. We selected the 100 top selling branded products in value, which account for 42 % (value) of the total market, in order to get a representative sample. Branded products are defined as the pharmaceutical products, whose active substance and trademark are under patent protection and no generics can be registered. We searched for the same products in the other countries, price was estimated based on defined daily dose (DDD) and, the final sample consists of 48 branded products indicated for primary care conditions in Portugal, Spain, Italy, Greece and Cyprus.

### Sources

We used the official price lists of the five  included countries. Moreover, personal communication with experts in each county, through the Pharmaceutical Pricing and Reimbursement Initiative(PPRI^1^)—supported by World Health Organization and Collaborating Centre for Pharmaceutical Pricing and Reimbursement Policies- were conducted for verification of data.

### Selected countries

Criteria for inclusion of comparative countries were defined in order to capture countries facing fiscal recession. Eligible countries were defined as those that experience financial recession, which is defined as two successive quarters of negative growth in gross domestic product. Criteria for inclusion were delineated as following:

- Introduction of measures to cope with financial deficit or gross debt, debt to GDP ratio of 60 %, and/or
- Adoption of Memorandum of Understanding on specific economic policy conditionality (or economic adjustment programme) with international lenders (International Monetary Fund, European Central Bank, European Commission –Troika) [8]

Comparative countries include Spain, Portugal, Greece and Italy and we searched for official retail prices of the same product across these countries. These countries are also relevant for Cyprus since they are among the basket of countries that Cyprus utilises for its external price referencing. Ireland exited the memorandum in 2013, nevertheless it was not included since pharmaceutical retail prices for private patients are not explicitly defined and its inclusion could have led to biased results [12].

A Laspeyer index was constructed from a Cyprus perspective. Laspeyrer index allows weight of data only for one participant, which in our case is Cyprus, and which will be used as the reference country due to availability of data. In this approach value and not price of basket products will define the significance of each product in Cyprus health care setting, in the elaboration of the comparative index. Laspeyer index captures in a more precise way the uptake and relevance of each product in a specific setting. Additionally, it depicts the significance in terms of budget impact and as such, it is superior to a price index. In this direction we used sales data from Cyprus private sector to weight each product with its actual budget impact. The use of retail price was preferred, in line with other author’s approaches, since this is the cost incurred to the patient [13, 14]. Moreover, ex-factory price is not applicable in Cyprus. We searched through official pricelists, as per 2011, of competent authorities in aforementioned countries, for which we selected the identical package and strength [15–19]. In case that the same package was not available across sample countries, price per DDD was assessed and the price was adjusted based on the recommended treatment duration (monthly for chronic disease) according to the formula adopted by Pharmaceutical services. Nevertheless, all products were available in the same package and strength.

In order to assess affordability, we weighted our findings on Gross Domestic Product (GDP) Purchasing Power Parity (PPP) per capita, a preferred tool for cross-country price comparisons [20, 21].

The second part of this study was performed to further confirm our findings. We compare affordability with impact of potential catastrophic pharmaceutical expenditure. We define the percentage of the population, that it will face catastrophic pharmaceutical expenditure after acquisition of one product from each major and common therapeutic category. One medicine was selected from each category, based on the prescribing pattern in Cyprus. We included products for the following conditions: diabetes mellitus type II, osteoporosis, blood pressure, hypercholesterolemia, asthma, dyspepsia (ulcer), depression and osteoarthritis. Data were extracted from Eurostat and we used minimum official wage of unskilled government worker from each country. For the definition of the catastrophic expenditure we used the rate of 5 % of household dispensable income, as per literature. We further analysed data for diabetes, as robust epidemiological data exist for this medical condition [22].

## Results

**Fig. 1:**
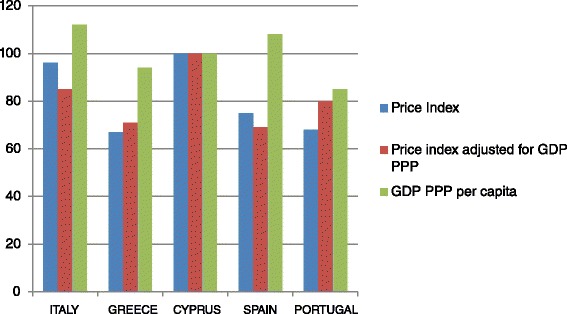
Graph of prices, GDP PPP per capita and adjusted price index for GDP PPP per capita in the selected Countries

The highest prices of private sector pharmaceuticals were reported in Cyprus and the lowest in Greece. After adjusting for GDP PPP per capita, Cyprus demonstrates the highest prices, followed by Italy and Portugal in the second and third place respectively (Table 3) (Fig. 1).

**Table 3:** Index of medicines’ prices and index adjusted by GDP PPP per capita

	Italy	Greece	Cyprus	Spain	Portugal
Index of medicines prices	95,52	67,4	100	75,36	68,99
Index of medicines adjusted by GDP PPP per capita	85,54	71,75	100	69,27	80,71

In order to assess our findings, we further verified them by defining impoverishment percentage. We assessed the impact of 8 products, one from each of the following major therapeutic categories: diabetes  mellitus type II, hypercholesterolemia, hypertension, depression, dyspepsia, Asthma, osteoporosis, anti-inflammatory (Table 4). Acquisition of one antidiabetic agent would be catastrophic for a 24 % of Italy’s diabetics patients, 25 % of Greece’s, 19 % of Cyprus’, 31 % of Spain’s and 23 % of Portugal’s (Table 5). In addition to this, acquisition of osteoporotic treatment would be financially catastrophic for Portuguese and Cypriots, while the acquisition of a combined medication for asthma would also be financially catastrophic for Portuguese and Cypriots patients.

**Table 4:** Affordability for eight major health conditions

Condition	Products	Strength	Price (Eur) (Price calculated per monthly basis for each denoted strength )				
			Italy	Greece	Portugal	Cyprus	Spain
Dyspepsia	Esomeprazole^a^	20 mg	7,08	10,81	8,8	18,63	6,3
Diabetes	Januvia	100 mg	59,22	50,22	50,53	58,37	55,72
Osteoarthritis	Etoricoxib^a^	90 mg	17,84	14,02	16,32	23,1	20,32
Blood pressure	Candesartan	16 mg	11,95	12,36	22,62	28,8	25,76
Hypercholesterolemia	Rosuvastatin	5 mg	22,49	11,27	17,01	28,74	18,9
Depression	Escitalopram^a^	10 mg	24,97	13,23	11,52	15,39	16,66
Osteoporosis	Zolendronic acid^b^	5 mg/100 ml	557,36	451,27	344,21	590,98	422,65
Asthma	Salmeterol/Fluticasone	25/50 μg	41,22	34,39	36,15	41,33	39,6
	Lowest monthly wage		992.4 [15]	878	565	870	748
	Catastrophic expenditure threshold		942.8	834	536	826	710

^a^For Esomeprazole, Etoricoxib and Escitalopram, the cost was calculated based on consumption for 28 days, thus calculating the cost of two packages

^b^For Zolendronic acid, the monthly cost was calculated, since this product is administered yearly

**Table 5:** Catastrophic health expenditure

	Italy	Greece	Cyprus	Spain	Portugal
Percentage of population of each acquisition of one product could be assessed as exceeding the threshold of catastrophic expenditure	24 %	25 %	19 %	31 %	23 %
Number of diabetics of which acquisition of one product could be assessed as exceeding the threshold of catastrophic expenditure	975,000	150,000	15,000	992,000	230,000

## Discussion

Access to necessary medicines is important and it becomes crucial during recession, since recession was proved to be an independent health risk factor leading to susceptibility for several health conditions [23]. Moreover, inability to finance current health needs does not cancel them out: it rather shift these costs to a later and costlier stage. Health agencies have implemented a series of measures to protect vulnerable groups. These include increase of reimbursement amount for pensioners as in Portugal, exception from user charges for low—income people and people with illnesses that would generate high out-of-pocket payments as in Italy, and reduced and capped co-payment rate in Greece and Spain. However, other policies shift patients to private sector in an increasing trend. Exclusion of unemployed citizens from public care as occurs in Spain, Greece and Cyprus, urges for caution since their buying power is severely compromised. Even indirectly, people are forced to resort to the private sector since austerity measures provide freeze of inclusion of new products in the formulary, reduction of formulary breadth, while shortages of reimbursed medicines take place regularly. Apparently, despite all assumptions trying to explain shortages, the leading reason is the accumulation of unpaid bills [24, 25]. In Greece, social insurance fund owes pharmacists 400 million euros, which directly impairs their liquidity and as result 203 products were withdrawn from Greece; among them 25 did not have generic available [26]. In Portugal, it is estimated that debts to industry have mounted to 1 billion euro and payments are on average 500 days due. Furthermore, in 2012, the combined accumulated debt of Greece, Italy, Spain and Portugal to the pharmaceutical industry exceeded 15 billion euro [27]. Consequently, the industry is reluctant to supply medicines due to accumulation of unpaid bills. As a result, suppliers accept only cash payment, which in many cases is not possible. This vicious circle end up at the patient, who is expected to pay in cash and procure the prescribed medicines as a private sector patient, an issue complicated by impoverishment and high unemployment rates. Therefore, eventhough, some products are officially reimbursed, are available only for private purchase.

Moreover, countries deliberately delay reimbursement process as a cost containment tool. In Portugal reimbursement period for some products may reach one year, while reimbursment decisions for specialised medicines may double that. Additionally, the approval of new drugs has ground to a halt for 18 months and reimbursement decisions have been freezed for non-generics, while the corresponding period for Greece spans up to 30 months. All aforementioned points attest to the fact that a significant percentage of the population depends on private sector for provision of healthcare.

Moreover, in Greece, persisting crisis has led to reduction of medicines intake by 33 % since patients cannot afford them. A 28.4 % of Greek patients space out their consumption due to affordability issues [28]. This was aggravated by the fact that Greece has not updated its positive list for 30 consecutive months, and all new products were available only in the private sector. In Italy, it’s estimated that the number of people that find it hard to buy their prescribed medicines increased two fold between 2006 and 2013 and the number of requests for medicines rose by 57 % [29].

Affordability is also relevant for publicly reimbursed products, for which patients incur a fixed amount or a percentage on the product's retail price in the form of co-payment. There are several reports entangling co-payment with affordability issues and a strong and immediate negative effect after increase of co-payment was documented [30]. Even more alarmingly, co-payment is more harmful for former good complying patients. While affordability reached the peak of its popularity as a demand proxy during financial crisis, several reports prior to crisis indicate that a significant percentage of patients (70.3 % in the UK and 66.5 % in Italy) expressed concerns about the cost of medicines, in terms of co-payment. Patients adopted several impromptu cost-containment approaches, such as selective purchase of prescribed medicines or delay or even avoidance to get any prescribed medicines at all [31]. Most interestingly, this study was performed in two high income European countries, UK and Italy, which both operate universal health coverage systems. Spain also experienced similar reduction in medicines’ consumption to a varying level across regions, which can be attributed to sensitivity of patients to new co-payment rates [32]. Therefore, we can speculate that situation is currently aggravated due to recession, consequent reduction of household expenditure and further dissemination of cost-sharing practices.

In view of the above, it is imperative that health agencies should try to explore other areas for cost containment and increase of income revenue. In all recession countries, the majorities of reforms that had to do with efficiency enhancement lagged expenditure cuts. Common practices such as electronic prescribing, HTA and guidelines have been applied at a later stage while cost reduction policies preceded. This raises question about procrastination and/or impotence of former health agencies to proactive apply measures [33] and it also displays the message of forethought policy making, especially during turbulent periods. Although, it is tempting to shift costs to patients, either through exclusion of beneficiary status, reduction of available products, or through disproportionate co-payment, this can backlash by hindering access of patients to necessary healthcare which can culminate in public health risk. Therefore cost reduction policies should be coupled with efficiency enhancement, eventhough the logistic impact of latter measures is highly uncertain.

Cyprus is in the worst position among the five assessed countries. The affordability of medicines, adjusted for GDP PPP per capita is the lowest among the 5 countries. Moreover, the acquisition of one product from three major categories (each one at a time) would be catastrophic for 19 % of its population. Cyprus, as one of the latest countries to apply for a bail-out, still has not implemented the magnitude of changes, as observed in the other countries. This results to high prices in the private sector, which given the impaired affordability of patients, can be considered as a potential restrictive barrier to pharmaceutical care. Moreover, the recent introduction of a prescription fee (1 euro) furthers aggravates current situation [34].

Medicines represent only a fraction of total health costs. Therefore, total financial burden on households is multiplied shall we include physician, laboratory and hospital costs.

Coverage during crisis must be universal. If this is not feasible, a safety net must be drawn for vulnerable groups. Patient’s access to effective medicines must be safeguarded at all costs. Private sector remains a realistic- or the only- option for a significant proportion of the population. Primarily, it should not be overlooked, under the flawed assumption that health systems offer a universal and adequate coverage. Financial burden should be equally distributed among involved parties, including pharmaceutical industry, patients and pharmacists. Pricing of pharmaceuticals is a step up process, spreading from ex-factory to gross retail, for that reason agencies should equally intervene on all stages of pricing. In some countries, such as Germany, wholesale price is only a small fraction of the final price, due to immoderate mark-up and pharmacist fee. In the majority of the cases, agencies regulate wholesale prices, since these are the officially defined prices and as a result fail to capture the magnitude of the problem. Therefore, retail prices, which incorporate general taxes as well that extend beyond pharmaceutical policy, such as VAT, must be scrutinized and monitored [35]. If deemed feasible, tax exemption must be considered as an alternative.

Innovative approaches must be introduced, targeting chronic patients, such as supply of subsided products through private distribution chain [36].

## Conclusion

It is well documented that satisfactory health levels must be considered as a goal to help a society overbear crisis and not as a target after crisis. Therefore, belt-tightening strategies should be adjoined with establishment of health as a social investment, with long-term focus. Agencies should proactively strive for efficiency instead of resorting to easy healthcare budget cuts, which may impair access of patients to the neccessary medicines. Access to affordable medicines must be safeguarded and affordability on essential healthcare should be assessed frequently. A focus on short-term financial gains is a myopic approach, which may increase inequity in health therefore health agencies should strive for long-term health and financial gains. Epidemiological studies must be carried out more frequently in order to detect earlier any trends which may demonstrate negative impact on public health. Since this is a primarily financial crisis, unless its root causes, such as corruption, bloated bureaucracy and tax evasion are identified and eradicated, all measures targeting the manifestations of the crisis will fail.

### Limitations

A laspeyer index was utilised for this study. Laspeyer index uses local weights of a specific setting (in our case Cyprus) to create a comparative index. One drawback is the possible failure to capture the exact patterns in other countries, since it is assumed that other countries follow the same pattern as Cyprus with regards to market share of pharmaceuticals used in the study. This could be erected as a barrier shall we compare countries with significant social and economic span. Nevertheless, we do not expect that significant deviations exist among pharmaceutical markets of these five countries, at least to the level that results are rendered unreliable.

## Abbreviations

DDD: Defined daily dose
EU: European Union
HTA: Health Technology Assessment
GDP: Gross domestic product
PPP: Purchasing power parity
VAT: Value added tax

## Footnotes

1 http://whocc.goeg.at.
